# Adolescent and young adult patients as co‐researchers: A scoping review

**DOI:** 10.1111/hex.13266

**Published:** 2021-05-15

**Authors:** Kjersti J. Ø. Fløtten, Ana Isabel Fernandes Guerreiro, Ilaria Simonelli, Anne Lee Solevåg, Isabelle Aujoulat

**Affiliations:** ^1^ Department of Integrated Care and Health Promotion Akershus University Hospital Lørenskog Norway; ^2^ International Network of Health Promotion Hospitals and Health Services (HPH) Taskforce on Children & Adolescents (HPH‐CA) Trento Italy; ^3^ Independent Consultant on Children's Rights in Health Care Albufeira Portugal; ^4^ Integrated Care Directorate Healthcare Trust of the Autonomous Province of Trento Trento Italy; ^5^ Department of Paediatric and Adolescent Medicine Akershus University Hospital Lørenskog Norway; ^6^ Department of Paediatric and Adolescent Medicine Oslo University Hospital Oslo Norway; ^7^ Institute of Health & Society UCLouvain Brussels Belgium

**Keywords:** adolescent patients, co‐research, health services, participation

## Abstract

**Background:**

As part of a research project aimed at evaluating a hospital‐based adolescent transition programme, we asked ourselves what is known about the ethical and methodological challenges of research involving adolescent patients as co‐researchers. The aim of our review was to summarize empirical evidence and identify knowledge gaps about the involvement of young patients as co‐researchers.

**Methods:**

We conducted a scoping review through searches in MEDLINE, EMBASE, PsychINFO, AMED.

**Results:**

We found reports of young patients being actively engaged as co‐researchers in any stage of a research project, although commonly they were not involved in every stage. Including young patients as co‐researchers is resource demanding and time‐consuming. Involving young patients as co‐researchers contributes to the fulfilment of their right to participation and may improve the relevance of research. Benefits for the young co‐researcher include empowerment, skills building and raised self‐esteem. Few authors go into detail about ethical considerations when involving young co‐researchers. None of the included articles discuss legal considerations.

**Discussion and conclusion:**

No lists of recommendations are given, but recommendations can be deduced from the articles. There is need for time, funding and flexibility when including young patients as co‐researchers. Knowledge gaps concern legal and ethical dilemmas of including a vulnerable group as co‐researchers. More reflection is needed about what meaningful participation *is* and what it entails in this context.

**Patient or Public Contribution:**

This review is part of a research project where the hospital youth council has been involved in discussions of focus area and methods.

## 1 INTRODUCTION

*‘The idea of citizen participation is a little like eating spinach: no one is against it in principle because it is good for you’*.^1^ What Arnstein pointed out in 1969 was that people were *talking the talk*, but were not necessarily willing to *walk the walk* of true citizen participation. It was a question of empty ritual versus real power to influence.^1^ Arnstein's original typology of the eight‐rung ladder of participation illustrates different forms of participation. The ladder is frequently referenced and has been adapted by many, also to health care.^2^, ^3^ It is now usually referred to by five levels: (a) information, (b) consultation, (c) advice, (d) collaboration and (e) control.^3^ Only the two last levels—collaboration and control—refer to approaches where power is truly shared.

We are seeing an increasing focus on patient participation in health research, with an increasing number of national guidelines being issued, for example in the UK,^4^ in Norway^5^ and in Belgium.^6^ However, as Malterud and Elvbakken point out, even though patient involvement in research has increased since the beginning of this decade, ‘*[…] this kind of research remains far from standard’*.^7^ Engaging patients in research on health care and health system issues is challenging for a number of reasons. Abma^8^ states ‘*social conditions for dialogue between patients and researcher are not given and should be sought for*’. In other words, in most cases the process does not spontaneously unfold. In addition, there are differences in the terminology used about participation and a need to clarify what concepts one uses. Abma^8^ argues that participatory approaches differ in their conceptualizations of participation, rationales of participation, norms and values, as well as their definitions of who is a legitimate participant. Participation at the level of consultation implies a different role to the role at the level of collaboration and control. The term ‘co‐researcher’ was coined by Smith in 1994 about being fully included in the research team,^7^ thus referring to the two upper levels of the participation ladder. There are lessons learned from projects that involved patients as co‐researchers, that is, with participation at the levels of collaboration and control. These include a need to clarify the roles of stakeholders, to reflect on what it means to be a patient representative, to choose the most suitable engagement methodologies for the project and to provide training and support to the patients so that they are sufficiently prepared. Furthermore, one should continuously monitor and provide feedback on the interactions within the project and be prepared to refine procedures as necessary.^9^, ^10^

As part of a research project evaluating a hospital‐based adolescent transition programme,^11^ we asked ourselves how the ethical and methodological challenges identified in research with adult co‐researchers apply when adolescent *patients* are co‐researchers. Children up to 18 years of age should be given the right to participate in research within the overall framework of human rights. Furthermore, the ladder of participation has been adapted also to children's participation.^12^ However, although some recommendations have been issued as part of the national INVOLVE initiative in the UK,^13^ there is little evidence of the benefits of partnerships with adolescents in research within health care. Indeed, a systematic review by Haijes *et al* ^14^ highlighted that participatory research in paediatrics is limited. Moreover, most results reported by Haijes *et al* ^14^ concern research projects where young people were consulted or allowed to give advice, thus referring to levels 2 or 3 of the participation ladder and not to co‐research. A recent scoping review by van Schelven *et al* ^15^ also points out that more research is needed to expand the evidence base of involving young people with chronic conditions in projects regarding their health and social care.

**FIGURE 1:**
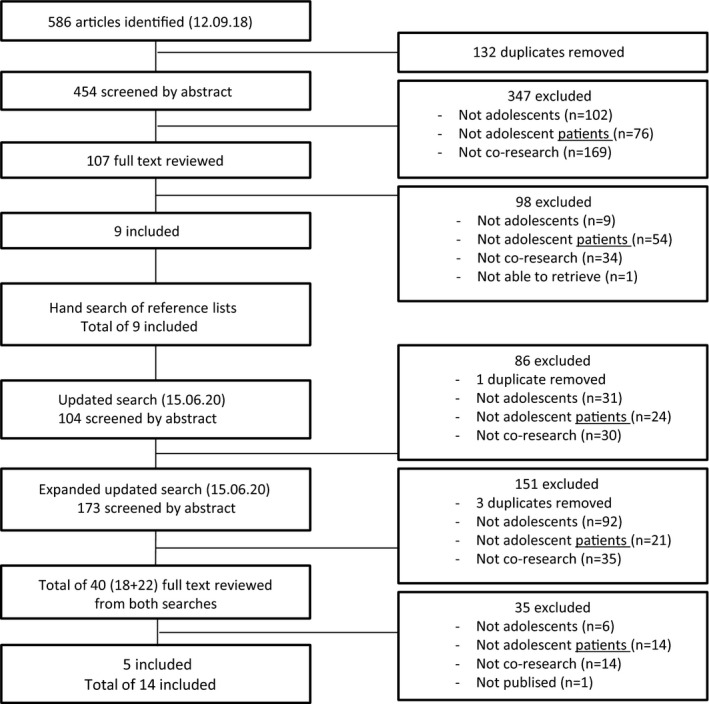
Flow chart of review process

The aim of this review was to summarize empirical evidence and identify knowledge gaps about the involvement of young *patients* as *co‐researchers*. By co‐research, we refer to full participation in the research team, that is levels 4 and 5 of the simplified participation ladder.^3^ Our review aimed to address the following questions: 1. At what stages of a research project are adolescent co‐researches engaged? 2. What methodological, legal and ethical issues and challenges are discussed? 3. What benefits and drawbacks for the co‐researchers, for the research and for other stakeholders are discussed? 4. What are the recommended strategies for meaningfully involving young patients as co‐researchers?

## 2 METHODS

To explore these topics, we performed a scoping review following the outline of the process as described by Peterson et al^16^ A scoping review gives an overview of a broad topic^16^, ^17^ and is a good method for exploring new topics.^16^

### 2.1 Data collection

A search of databases (Table 1) was performed in September 2018 with the help of a medical librarian. The following databases were searched: MEDLINE, EMBASE, PsychINFO and AMED. The search was limited to articles written in English, Norwegian, Swedish, Danish, French, Italian, Portuguese and Spanish. This yielded 454 results. The search in AMED yielded no results and is therefore not presented in the table.

**TABLE 1:** Overview of search string

Database	Search string
EMBASE Ovid MEDLINE(R) ALL PsycINFO	*Child/OR (child or children).ti,kf,kw,id. OR *Adolescent/OR (adolescent* or youth* or teenager* or teen* or young people).ti,kf,kw,id. AND *Community‐Based Participatory Research/OR *participatory research/OR *action research/OR co‐research*.ti,ab,kf,kw,id. OR participatory research.ti,ab,kf,kw,id. OR participatory action research.ti,ab,kf,kw,id. OR ((child or children or adolescent* or youth* or teenager* or teen* or "young people") adj15 (co‐research* or "participatory research" or "action research")).ab. AND exp *Hospitals/OR hospital*.ti,kf,kw,id. OR exp *Health Services/OR (health service* or health care setting*).ti,kf,kw,id. limit to (danish or english or french or italian or norwegian or portuguese or spanish or swedish) limit 22 to yr="2000 ‐Current" remove duplicates

In stage two of the process, the first and last author screened the abstracts. The following inclusion criteria were used: articles concerning patients 12 years and above; patients are co‐researchers; the research topic is in relation to health care and health systems research; describes 4th and 5th level of participation according to the participation ladder as amended by Teunissen and referenced by de Wit *et al* ^3^; and publication date 2000–current. The first author and one co‐author performed the full‐text review. In case of disagreement, a third author was consulted. All co‐authors were asked to consider whether they knew other publications that should be reviewed. The reference lists of the included articles were hand‐searched. This did not lead to any further inclusions. The search was updated in June 2020, and an additional 5 articles were included. Thus, we included 14 articles out of 731 (454 + 277) (Figure 1).

The characteristics of the included articles are presented in Table 2.

**TABLE 2:** Characteristics of studies

Author(s):	Dunn, 2017	Edwards et al, 2016	Flicker, 2008	Flicker et al, 2005	Kruzich & Jivangee, 2011	Lincoln et al, 2015	Mitchell et al, 2017	Valencia et al, 2010	van Staa et al, 2010	Cleverley et al, 2020	Kramer & Schwartz, 2018	Moules, 2009	Pullmann et al, 2013	Wintels et al, 2018
Language:	English	English	English	English	English	English	English	Spanish	English	English	English	English	English	English
Country of study:	UK	UK	Canada	Canada	US	US	Canada	Colombia	Netherlands	Canada	US	UK	US	Netherlands
Aim:	To coproduce a transition preparation programme.	To develop a framework for a service evaluation measure for young people to use.	To explore who benefits from community‐based participatory research in health research.	To investigate what can be done to better support HIV‐positive youth	To explore perceptions of barriers and facilitators for community integration of young people with mental health needs. To influence design of an intervention.	To gain understanding of housing support needs of Transition Age Youth living with mental health conditions. To develop a mechanism for this group to take part in design and conduct of mental health research.	To gain understanding of experiences of young adults in Take Home Naloxone (THN) programmes. To get young adults suggestions for improvement of such a programme.	To identify perceptions, experiences and expectations about health services for youth and health‐care agents from the Zona de Ladera in the city of Cali. To develop a strategy to improve health services under the ‘Friendly Youth Services’ (SAJ abbreviation in Spanish) guide.	To evaluate a participatory research (PR) project involving chronically ill adolescents as ‘co‐researchers’	To explore qualitatively the experiences of youth transitioning from Child and Adolescent Mental Health Services to Adult Mental Health Services	To develop the Pediatric Evaluation of Disability‐Patient Reported Outcome (PEDI‐PRO) for 14‐21‐year‐olds	To explore quality of care in hospital	To develop conceptual definition of engagement in adolescent substance abuse services	Explore participation experiences of adolescents with cerebral palsy (CP)
Research design/methods (as labelled by authors):	Participatory Research	Qualitative mixed methods	Qualitative approach to data	Community‐based participatory research	Participatory action research	Community‐based participatory research	Principals of community‐based participatory action research	Action research	Participatory research	Participatory action research	Participatory research approach	Participatory study	Participatory action research	Participatory research design
Description of participants:	Age 17‐22 Total of 18	Age 17‐25 Total of 12	Total of 79	Unspecified number of HIV‐positive youth took part in stakeholder group	Age 17‐24	Age 18‐25	Two peer researchers with lived experiences	Age 10‐19 Total of 100	Age 15‐17 Total of 9	Age 16‐18	Team of 8 youths with DD, Age 14‐21	9 youth Age 12‐16	3 youths Age 17‐19	12 ambassadors

### 2.2 Quality of reporting

Although not mandatory for a scoping review, we chose to perform a quality assessment of the included articles. As argued by van Schelven *et al,* it helps to put the results in context.^15^ The quality of reporting was assessed by the last author. Assessment of the article in Spanish was done by a Spanish‐speaking co‐author. The quality appraisal was intended as indicative and was not part of the selection process. A protocol designed by Kmet et al^18^ was used, which includes ten items. Details are outlined in Table 3. Each article was assessed according to the fulfilment of items and was rated as ‘yes’ (=2), ‘partial’ (=1) or ‘no’ (=0) on each item. Each study was assigned a summary score between 0 and 1 (with higher scores indicating better quality of reporting) by adding the scores of all individual items and dividing by the maximum possible score (20 for qualitative studies).

**TABLE 3:** Quality of reporting

	Dunn, 2017	Edwards et al, 2016	Flicker, 2008	Flicker et al, 2005	Kruzich & Jivangee, 2011	Lincoln et al, 2015	Mitchell et al, 2017	Valencia et al, 2010	van Staa et al, 2010	Cleverley et al, 2020	Kramer & Schwartz, 2018	Moules, 2009	Pullmann et al, 2013	Wintels et al, 2018
Question/objective sufficiently described?	2	2	2	2	2	2	2	2	2	2	2	2	2	2
Study design evident and appropriate?	2	2	2	2	2	2	2	1	1	2	2	2	1	2
Context for the study clear?	2	2	2	2	2	2	2	2	2	2	2	1	2	2
Connection to a theoretical framework/wider body of knowledge?	1	1	2	1	2	2	2	2	1	2	2	1	2	1
Sampling strategy described, relevant and justified?	2	1	1	2	2	2	2	2	1	2	1	2	1	2
Data collection methods clearly described and systematic?	2	2	2	2	2	2	2	1	2	2	2	1	1	2
Data analysis clearly described and systematic?	2	2	2	2	2	2	2	2	0	2	2	2	2	2
Use of verification procedure(s) to establish credibility?	1	1	2	2	2	2	2	1	1	2	2	1	2	2
Conclusions supported by the results?	2	2	2	2	2	2	2	2	2	2	2	1	2	2
Reflexivity of the own account?	1	1	2	0	1	1	1	1	1	1	2	1	0	1
Total score per article	0.85	0.8	0.95	0.85	0.95	0.95	0.95	0.8	0.65	0.95	0.95	0.7	0.75	0.9
	Criterion fulfilled (score 2)	Partially fulfilled or don't know (score 1)	Not fulfilled (score 0)											

### 2.3 Data analysis

An extraction grid was developed. Categories were created to reflect the research questions and the stages of a research process. The extraction grid was tested by the first and last authors. Amendments were made before application to the included articles. An integrated form of analysis was performed^19^ with the help of the extraction grid.

## 3 RESULTS

### 3.1 Characteristics of the included studies

None of our 14 articles were included in the review by Haijes & van Thiel.^14^ Two overlap with those included by van Schelven *et al* ^15^ Four articles were from the mental health context.^20^, ^21^, ^22^, ^23^ Three reports included patients with somatic conditions followed up in hospital.^24^, ^25^, ^26^ Eight articles were from Northern America, three from the UK, two from the Netherlands and one from Colombia. Twelve projects included adolescents aged 17 years and above. Kramer and Schwartz^27^ included co‐researchers aged 14‐21, and Moules^26^ had co‐researchers as young as 12‐16 years of age. All studies reported on projects with a mainly qualitative approach.

### 3.2 Quality of reporting

Quality assessment scores ranged between 0.65 and 0.95. All scores but three were over 0.8, which indicates a high quality of reporting for most articles (Table 3).

### 3.3 Participation may happen at any stage, but seldom at every stage

There is evidence of adolescent co‐researchers being involved in all six stages of research (Table 4), but only two projects report on co‐researchers being part of all stages.^20^, ^22^ In the **preparatory phase,** there is focus on research training. Van Staa *et al* ^24^ did a field trip with the co‐researchers to a newspaper to gain insight and discuss the interview protocol, while Dunn^20^ reports on formal literature search training. Some do not go into details on the training given^26^, ^28^, ^29^ while Lincoln and colleagues^22^ report on extensive training and formal testing of knowledge. It is argued that research training addresses the imbalance of power between researcher and co‐researcher and that the training provides the adolescents with a wider knowledge base.^25^

Four articles report on involvement in the **recruitment phase**. This is the area with least reported participation. These articles report on co‐creation of the recruitment tool^21^ or participation in design and dissemination of the recruitment material.^20^, ^30^ Lincoln *et al* ^22^ report that the recruitment strategy was discussed by ‘the whole research team’. Most articles report on involvement in the **design phase** including project development meetings,^25^ protocol writing,^30^, ^31^, ^32^ decision on methods,^22^ discussions of research questions,^26^, ^29^ development of questions for interviews, focus groups or preparation of workshops.^20^, ^21^, ^23^, ^24^, ^26^, ^27^, ^28^, ^29^ Nine articles report on participation in the **data collection phase**. Young researchers were involved in data collection such as interviews or focus groups.^20^, ^21^, ^22^, ^24^, ^25^, ^26^, ^27^, ^28^, ^29^ In the case of focus groups, they either led or co‐hosted the group.

All articles report on involvement in the **analysis phase**. Some report briefly that the analysis was done by the team,^21^, ^23^, ^26^, ^27^, ^29^ that adolescents were involved in ‘*continuous review of all work’* ^25^ or that the results were discussed.^33^ Others are more specific, for example describing that the co‐researchers transcribed their own interviews after which the group as a whole performed the analysis.^22^ Mitchell *et al* ^28^ describe a process of sharing transcripts and analysing the data as a group. Wintels *et al* ^31^ write that adolescents were involved in coding and member check. Flicker *et al* ^32^ report on weekly stakeholder group meetings for discussions. When it comes to the **dissemination stage,** some simply state that dissemination was done ‘*as determined by the team’* ^22^ or was shared.^28^ Others report co‐presentations in different forums, including co‐design of conference posters or conference presentations.^29^ Co‐writing publications ranged from co‐writing the published article^23^, ^25^ to popular or ‘unspecified’ publications.^20^, ^24^, ^30^

### 3.4 Methodological, legal and ethical issues and challenges

The reasoning behind choice of methods revolves around the importance of involvement to better meet the needs of adolescents^21^ and arguing the need to do research with rather than on patients.^20^, ^24^, ^27^ It is argued that participatory or qualitative methods are suitable because they give rich opportunities for adolescents to voice their opinion and share their experience.^21^, ^22^, ^23^ Challenges of participatory methods are raised when it comes to having co‐researchers follow all steps of the research process. Van Staa *et al* ^24^ struggled with co‐researcher engagement in the analysis stage. Others such as Edwards *et al* ^25^ had some adolescents that followed all stages, while others took part in a selection of stages. The additional burden that participation puts on young and sick co‐researchers is problematized.^24^ Lincoln *et al* ^22^ also address the challenge of defining who ‘the community’ in a community‐based participatory research approach actually is, thus problematizing representativeness of the co‐researchers.

**TABLE 4:** Simplified representation of extraction grid

Stage of involvement^a^	Dunn, 2017	Edwards et al, 2016	Flicker, 2008	Flicker et al, 2005	Kruzich & Jivangee, 2011	Lincoln et al, 2015	Mitchell et al, 2017	Valencia et al, 2010	van Staa et al, 2010	Cleverley et al, 2020	Kramer & Schwartz, 2018	Moules, 2009	Pullmann et al, 2013	Wintels et al, 2018
Preparatory work	Responding to demographic questionnaire that informed workshop design. Literature review training.	Providing a wider knowledgebase.	Early inclusion in working group. Training in research method.		Two research assistants. Members of local advisory group.	Involved in research team. Extensive research training Literature review.	Two peer researchers recruited. Research training with co‐investigators.		Co‐researchers invited through nurses. Introduction to research techniques.			Interviews done with co‐researchers. Discussions on progress. Methods training.	Literature review. Training in methods.	
Recruitment	Through young peoples' participation network.		Design and distribution of material.		Recruitment tool.	Strategy discussed by team.								
Design	Consulted on design and topic. Co‐design of clinician workshop.	Through project development meeting.	Design of protocol.	Stakeholder group co‐created protocol.	Development of pre‐focus group questionnaire. Focus group questions.	Development by research team. Research questions. Decision on method.	Shared decision making. Development of semi‐structured interview guide.		Preparation of interview protocol through discussion of drafted protocol.	Reviewed interview guide.	Develop focus group.	Deciding on research questions. Vignette construction.	Discussion of research questions. Discussion of focus group questions.	Gave input on protocol. Discussion on content of interviews.
Data collection	Co‐host workshops clinicians. Part of creative participatory workshops.	Workshop.			Young research assistant led focus groups.	Performed interviews. Transcribed interviews.	Shared Focus groups. Individual interviews.		Performed interviews.		Collected data from own life (written, photo, video and field note). Co‐facilitated focus groups.	Took part as storytellers of vignettes.	Took part in focus groups.	
Analysis	Process of consensus in workshop. Possibility to feedback on draft synthesis.	Continuous review of all work.	Invited back for analysis. Consensus process.	Weekly meetings of stakeholder group for analysis.	Done by the whole team.	Team performed the analysis.	Read transcripts and analysed data as group.	Involved in discussion of results.	Invited to discussion but unable. Consulted on draft through email.	Participated in team.	Analysed as a group.	Analysed interviews. Analysed vignette data.	Done by the whole group.	Involved in coding. Member check.
Dissemination	Co‐design of conference poster. External and internal dissemination. Co‐authoring an article.	Co‐authoring the article.	Publications. Zines Taking part in community‐wide forum	Co‐authoring article.		‘*As determined by team*’ – not specified.	‘*Shared*’ – Not specified.	Co‐develop strategy. Joint design of space. Recommendations to management.	Media activities f.ex. interviews. Popular article. Invited to conference	Participated in writing up publication.			Conference presentations.	

^a^ Amended from de Wit, M., Beurskens, A., Piškur, ES. & Moser, A. (2018) *Preparing researchers for patient and public involvement in scientific research: Development of a hands‐on learning approach through action research* Health Expectations. 1‐12. John Wiley & Sons Ltd.

Few authors go into detail on ethical considerations. Most state that an ethics committee evaluated their project. Safeguarding confidentiality and informed consent is touched upon. Lincoln *et al* ^22^ describe ethical considerations when developing what they call a human research protocol and that the institutional review boards raised questions related to, for example, the ability of the young co‐researcher to judge the informant's capacity to consent, and whether the young researcher could be faced with their own personal problems while interviewing. To address this, the research team developed a self‐care plan. Flicker^30^ addresses the risk of disclosure and stigma that a young co‐researcher faces, that is, that by getting involved as co‐researchers they also make public their lived experience of the addressed challenge. No article discusses legal challenges or the role of parents in co‐deciding or giving permission for participation as co‐researchers.

### 3.5 Benefits and drawbacks of involving young patients as co‐researchers

Benefits for the young co‐researchers, the professional researcher and the research process are discussed. Arguments used are primarily for the research itself, including relevance of questions, methods and findings, as well as recruitment.^22^, ^23^, ^25^, ^27^, ^30^, ^31^, ^32^ Benefits for the young co‐researcher include empowerment, skills building and raised self‐esteem.^24^, ^30^ The amount of time and the resources required from the participative research processes are problematized.^24^, ^29^, ^30^ Van Staa *et al* ^24^ question whether participation automatically adds value. Lincoln *et al* ^22^ raise the challenge that disclosing one's membership to a community might lead to stigmatization. Getting ethics boards to understand what participatory research entails^22^ was pointed out as another challenge. The discussion of benefits and drawbacks is more reflections on the participation processes, than a report of structured evaluations of the process.

### 3.6 Recommended strategies for meaningful involvement

No article draws up a list of recommendations, but the importance of clarifying roles has been emphasized,^30^ especially as this may be an unfamiliar situation for both adult researchers and young co‐researchers. Adequate funding is also important^30^ as these are processes that are generally more time and resource demanding than more traditional forms of research. A second recommendation is to address, in advance, questions of who will benefit from the research.^24^, ^30^ There are three particular recommendations to safeguard the interests of the young co‐researcher. The first is proper training.^25^ The second is to develop a self‐care plan so that possible challenges are discussed and addressed ahead of time.^22^ Finally, it is recommended to be flexible, that is, that the young co‐researchers should be made aware of the possibility to step out of the research, and/or choose which stages of the research to participate in.

## 4 DISCUSSION

This is the first scoping review of adolescent *patients* participating at level 4 or 5 according to the simplified ladder of participation. We identified only 14 articles matching the inclusion criteria. We find it interesting that we do find evidence of young co‐researchers being part of all stages of the research process. This is a similar to findings reported in the review by Van Schelven *et al* ^15^ However, what topics are reported on still raises a number of questions for further discussion.

### 4.1 Ethical issues

It is our opinion that the included articles would have benefited from a more in‐depth portrayal of ethical challenges of involving young co‐researchers. This would have provided important learning points for other researchers. Most mention that they have gone through ethics committee approval, but with one exception,^22^ they do not go into detail about what issues they highlighted in their reporting, nor what concerns the committees raised in their responses. Gilchrist *et al* argue ‘*Ethical issues to be considered when carrying out research or service evaluation with children may include: power relationships, consent issues, confidentiality and dissemination of results’*.^34^ They acknowledge that these issues are not unique to research with children (meaning up to 18 years of age), but argue that there may be a need to pay particular attention to them in this context. Furthermore, Gilchrist and colleagues^34^ state that we live in an adult‐centred society and that the power adults have can be carried over into research. This is an argument also made in a literature review by Kirk.^35^ In health care, this is influenced by the authority that adolescents are used to impart to their health‐care providers. They are used to assigning themselves a subordinate role as patients and of lesser knowledge and age. Whether adolescents and their health‐care providers are used to seeing young patients as valid knowledge holders in the context of research will play an important role in their involvement. Are they judged by health professionals as mature enough to take part?^36^ Or are the rights to participation lagging behind because health professionals are used to thinking in terms of the right to protection and that safeguarding the best interest of the child is more or less equivalent to protection?^36^ The power relation between young patient and health professional is such that in most cases the adolescent would need an invitation from the adult researcher to join research. They would most likely not be the one to take the initiative. Thus, it is essential that the adult researcher see participation in research as relevant. These ethical considerations should be discussed to advance participation.

A related issue is research ethics boards' project assessment. How can we ensure that they properly assess not just the research process the co‐researchers are to be involved in, but also the role as co‐researcher? Researchers wanting to involve young co‐researchers should consider and have to account for ethical questions suitable for their projects. This was exemplified by Lincoln *et al* who developed a self‐care plan for the young co‐researchers' well‐being.^22^ Similarly, Kirby^13^ highlights the importance of recognizing that taking part in research may impact on emotional well‐being and that one should discuss this with the young co‐researchers at an early stage. The INVOLVE guide^13^ also addresses issues of safety and well‐being when adolescents take part in fieldwork.

### 4.2 What rungs of the ladder do we aim for?

The discussion on ethics ties into reflections we should have when utilizing the ladder of participation. It is easy to get the impression that participation at the higher rungs of the ladder should be preferred. However, is this really the case? Hart states that his adaptation of the participation ladder, contrary to how it has been used by some, was not meant to be an evaluation tool. He intended his adaptation to be used as a way of portraying different forms of participation and as a point of departure for reflexion.^12^ Are there situations where we should *not* aim for the top rung of the ladder, for example if aiming for the 5th level of the ladder entails leaving the adolescents on their own in a research process where they should have support? Jones argues that a healthy work environment is the responsibility of the adult researcher.^37^ Furthermore, she argues that this should be safeguarded in the process where one considers barriers and boundaries of participation in research. In this process, one should not only look at what barriers to overcome, but also what boundaries to establish for the safety of the adolescent co‐researcher.^37^ Furthermore, Hart states that ‘*It is not appropriate that some children feel that they must always only follow the initiative of others any more than it is good for any child to feel that they should always be a leader*’.^12^

### 4.3 How do we prepare participants for co‐research?

A question that arises after reviewing the included articles is what is relevant or adequate research training for young co‐researchers? It has been suggested by others that fully involving children as co‐researchers can be problematic due to their lack of the theoretical knowledge needed.^35^ The way research training was approached, differed greatly in the reported projects. Therefore, the included articles do not provide a clear roadmap for other researchers to follow. The larger the training package, the more time and resource demanding the process will be. This is not an argument against involvement and training of the adolescents, but an argument for thinking this through and accounting for it before endeavouring on such a process. There is a fine balance between providing research training to the extent that the adolescents feel comfortable to take part and ‘demystifying’ research for them, while still preserving their commitment to the required time and effort.^38^ Interestingly, few of the included articles problematizes what kind of training professional researchers might need to endeavour into co‐research with young patients.^12^

It is important to keep in mind that research training is not the only way to tackle the issue of power balance in the research group. This could also be addressed through creating spaces and opportunities for meaningful participation,^39^ giving each other room, being curious and valuing different kinds of knowledge. One of the most valuable insights that can be gained from adolescent co‐researchers is their lived experience as patients, of the care they received and the impact their illness has had in their lives. This can benefit the research in what focus it takes and what questions it investigates. Abma argues ‘*People understand the world in different ways but we do not know what those differences are until we have an opportunity to share and discuss’*.^40^ If we are to tease out the benefits of participatory research, we need to acknowledge that academic knowledge does not have primacy over other forms of knowledge.^40^ Creating a space where other forms of knowledge emerge may be more important than expecting academic rigour from the young people involved. In fact, Jones argues that children (up to the age of 18 years of age as per the CRC) cannot be held responsible for research,^37^ this responsibility lies with the adult researcher. Research should take into account the skills of the person facilitating the interviews, focus group and overall participation process. Even with education and training, we cannot expect young co‐researchers to inhabit the same skills as an experienced adult researcher.

The question remains what all this entails for the training of young co‐researchers. We find the example of Jones^37^ interesting. She reports on a project where the training involved learning not only about research methods, but about protecting the rights of the researched, how to ensure own health and safety, techniques to use in interviews, how to logistically get to interviews, planning for things that can go wrong, how to record interviews and also how to do preliminary data analysis.^37^

### 4.4 Challenges and drawback

Van Schelven *et al* report in their review that some of their included articles problematize that lack of research experience can lead to lack of depth in for instance interviews.^15^ This is also a concern raised in the article by Van Staa *et al* included in this review.^24^ Again, we see the need for a discussion on what the top rung of the participation ladder and co‐research really entails. Does it mean that young co‐researchers take complete lead and are left on their own in all stages of the project? Or, is it more fruitful to think in terms of partnership where the experienced researcher and the young co‐researcher, and the research for that matter, would benefit from partnering in the data collection? It all comes back to what the goal of the process is. Is it as Hart puts it liberation of the young co‐researcher from adults, or is it to recognize the rights of others to participation and therefore involve them?^12^

### 4.5 Learning points for future research projects

Finally, what can be learned from the included articles is the need for flexibility. The need for flexibility was also pointed out by van Schelven and colleagues.^15^ A young person may not be able to commit for a long period for different reasons. This brings us back to the discussion of what we aspire to achieve through the participatory process. Is it to have the same adolescents follow the whole process or is it having *someone* follow each step? And if adolescents are invited to full participation, are well informed, but opt for a lower level of participation such as being consulted, does that mean that the project is not truly participatory? We argue that research would benefit from a mix of forms of participation.^36^ Young people themselves state that there is not one ‘right’ way of involvement, because preferences vary from person to person.^41^ Similarly, the INVOLVE guide cautions against assuming that young people cannot be involved in certain stages of the research, but also against assuming that they wish to be involved in all the stages of the research.^13^ Furthermore, there is a need to acknowledge that desire to participate might shift through time.

### 4.6 Strengths and limitations

One limitation of the review could be specific databases indexing that may have prevented us from identifying relevant publications. Van Schelven *et al* ^15^ problematize that there is a lack of conceptual clarity of PPI resulting in different definitions of the same concepts. This could also have contributed to potential challenges in identifying all relevant studies.

## 5 CONCLUSION

No lists of recommendations are given in the included articles, but recommendations can be deduced from the articles. There is need for time, funding and flexibility when including young patients as co‐researchers. We would argue that research involving adolescents co‐researchers should be based on and would benefit from proceeding in line with a rights based framework. We miss a more thorough discussion of legal and ethical dilemmas when including a particularly vulnerable group as co‐researchers. More reflection is needed about what meaningful participation *is* and what it *entails* in the context of research. Proceeding in line with a child/human rights framework could form the basis for adopted recommendations on how to involve young co‐researchers, as well as the development of self‐care plans. Research ethics boards need the competencies to properly assess not just the research process that the adolescents are to be involved in, but their role as co‐researchers.

## CONFLICT OF INTEREST

The authors declare no conflict of interest.

## AUTHOR CONTRIBUTIONS

Kjersti J. Ø. Fløtten designed and conducted the work presented in this paper, analysed the data and wrote the paper. Isabelle Aujoulat designed the work, analysed the data and has co‐written the final version of the manuscript. Ana Isabelle F. Guerreiro has participated in the design, offered advice on data collection and has co‐written the final manuscript. Ilaria Simonelli has participated in the design, offered advice on data collection and has co‐written the final manuscript. Anne Lee Solevåg participated in the design, offered advice on data collection and has co‐written the final version of the manuscript.

## Data Availability

Data sharing is not applicable to this study as no new data were created or analysed.
